# Supplementary material for: Cell stress and phase separation stabilize the monomeric state of pseudoisocyanine chloride employed as a self-assembly crowding sensor
Source: Commun Chem. 2024 Oct 7;7:230. doi: 10.1038/s42004-024-01315-y (PMC11458801; doi:10.1038/s42004-024-01315-y)
Supplement: Supplementary file 2 — Supplementary Information [file 42004_2024_1315_MOESM2_ESM.pdf]

Supplementary Information (SI)

Cell stress and phase separation stabilize the monomeric state of pseudoisocyanine chloride employed as a self-assembly crowding sensor

Roland Pollak[a,b], Leon Koch[c], Benedikt König[a], Sara S. Ribeiro[a,b], Nirnay Samanta[b,†], Klaus Huber[c,*], Simon Ebbinghaus[a,b,*]

[a] R. Pollak, S.S. Ribeiro, Dr. B. König, Prof. Dr. S. Ebbinghaus, Lehrstuhl für Biophysikalische Chemie, Ruhr-Universität Bochum, 44780 Bochum, Germany and Research Center Chemical Sciences and Sustainability, Research Alliance Ruhr, 44780, Bochum, Germany, Universitätsstraße 150, D-44801 Bochum, Germany, E-Mail: simon.ebbinghaus@rub.de

[b] R. Pollak, S.S. Ribeiro, Dr. N. Samanta, Prof. Dr. S. Ebbinghaus, Institut für Physikalische und Theoretische Chemie, TU Braunschweig, Rebenring 56, 38106 Braunschweig, Germany,

[c] L. Koch, Prof. Dr. K. Huber, University Paderborn, Institute of Physical Chemistry, Warburger Straße 100, D-33098 Paderborn, E-Mail: klaus.huber@upb.de

Present addresses

† Dr. N. Samanta, Department of Biochemistry and Molecular Biophysics, Washington University in St Louis, Saint Louis, Missouri 63110 United States

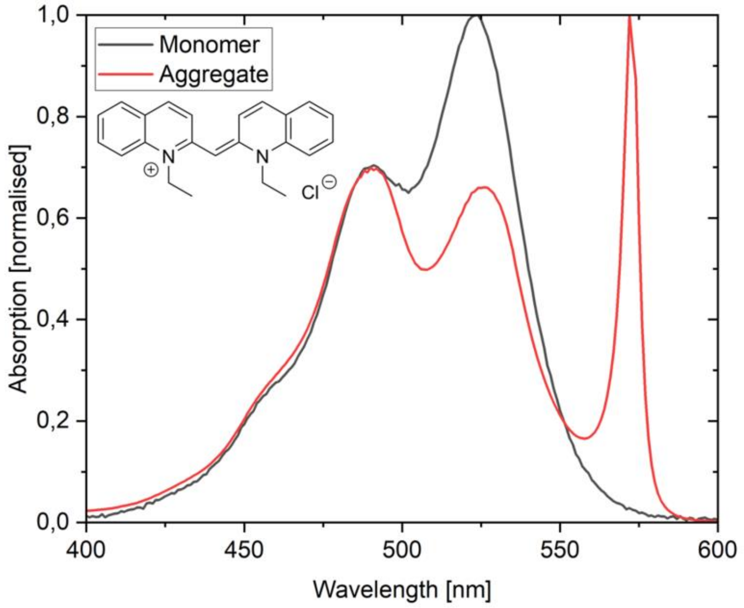

Supplementary Fig. 1. Absorption spectra of PIC in aqueous solution. The monomer (100 µM, black) shows a main peak at 523 nm and a 2nd peak at 491 nm. Beyond the aggregation threshold (8 mM, red), a sharp peak at 573 nm, characteristic of the J-aggregate, is observed.

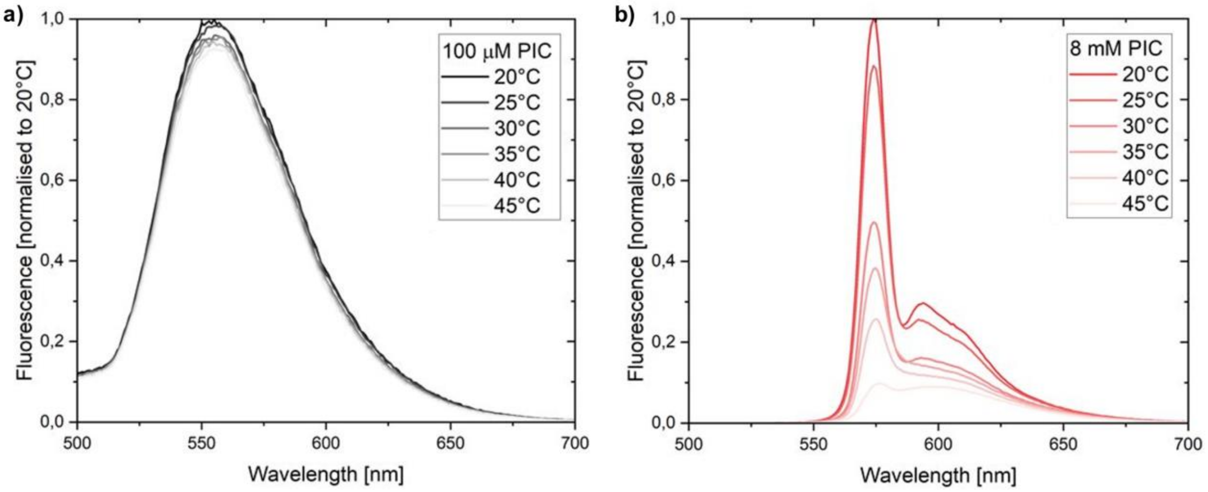

Supplementary Fig. 2. Temperature-dependent fluorescence spectra of PIC in aqueous solution. a) The fluorescence of monomeric PIC (black) appeared as a broad spectrum from 500 nm to 700 nm with a peak at around 555 nm and reduced fluorescence at higher temperatures. b) The sharp fluorescence peak of the J-aggregate (red) was located at 575 nm. The shoulder at higher wavelength could be assigned to smaller PIC multimers. The aggregate solution showed a strong fluorescence decrease with increasing temperatures. Furthermore, the J-aggregate peak intensity at 575 nm decreased to the shoulder level at higher temperatures, suggesting that the J-aggregates break into smaller multimers with increased temperature.

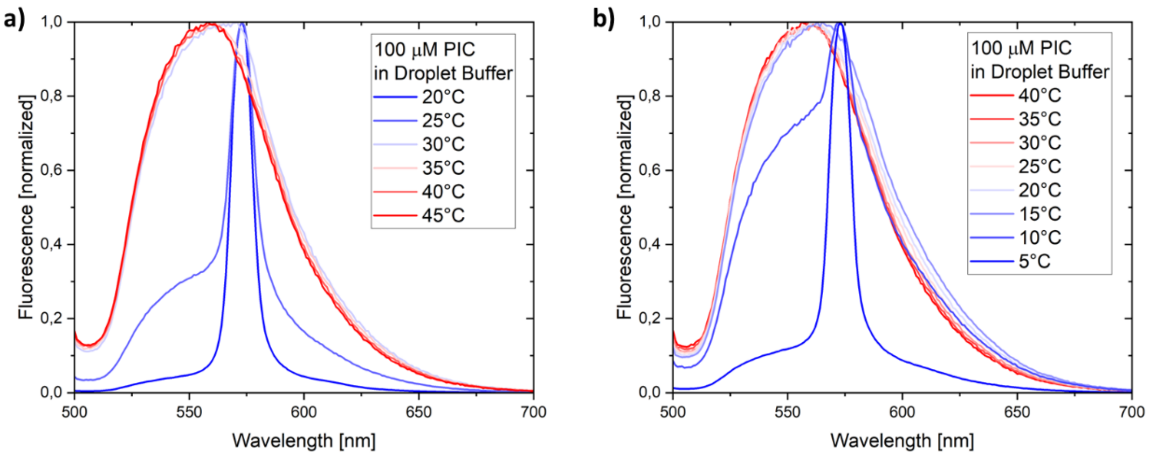

Supplementary Fig. 3. Temperature-dependent fluorescence spectra of PIC in droplet buffer. a) The fluorescence of the J-aggregated PIC at 20°C transitions into the monomeric PIC fluorescence with rising temperature (20 to 45°C). b) The same solution was then cooled down, which resulted into the reformation of the J-aggregate.

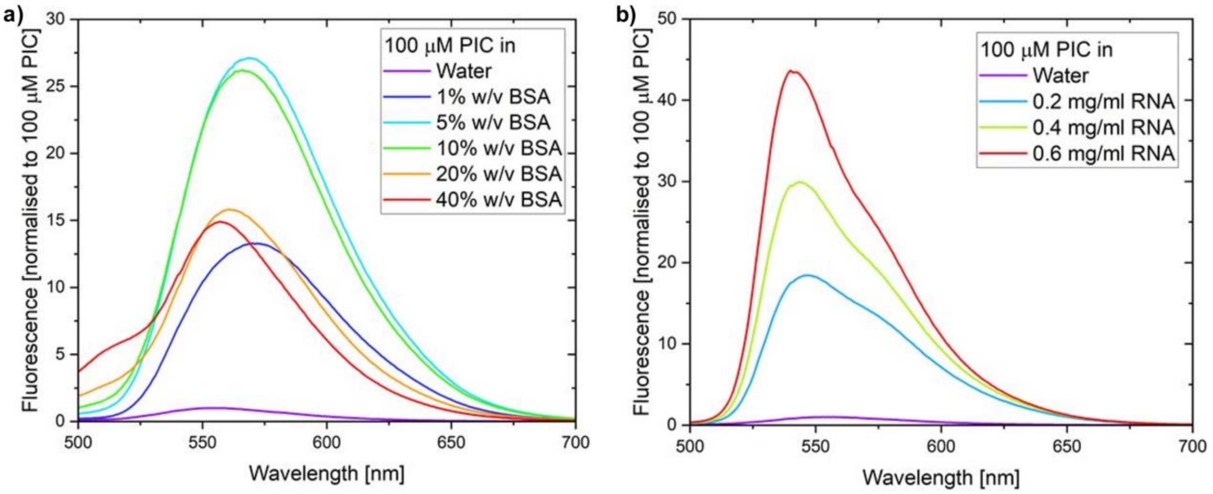

Supplementary Fig. 4. Spectra of monomeric PIC in aqueous solution with BSA and total RNA. a) The fluorescence of monomeric PIC (purple) was strongly increased with added BSA. While the fluorescence increased to a maximum at 5% w/v BSA, a further increase in concentration led to a decrease in fluorescence. A shoulder appeared at 510 nm with increasing BSA concentration. b) The fluorescence of monomeric PIC (purple) was strongly and linearly increased with added total RNA concentration.

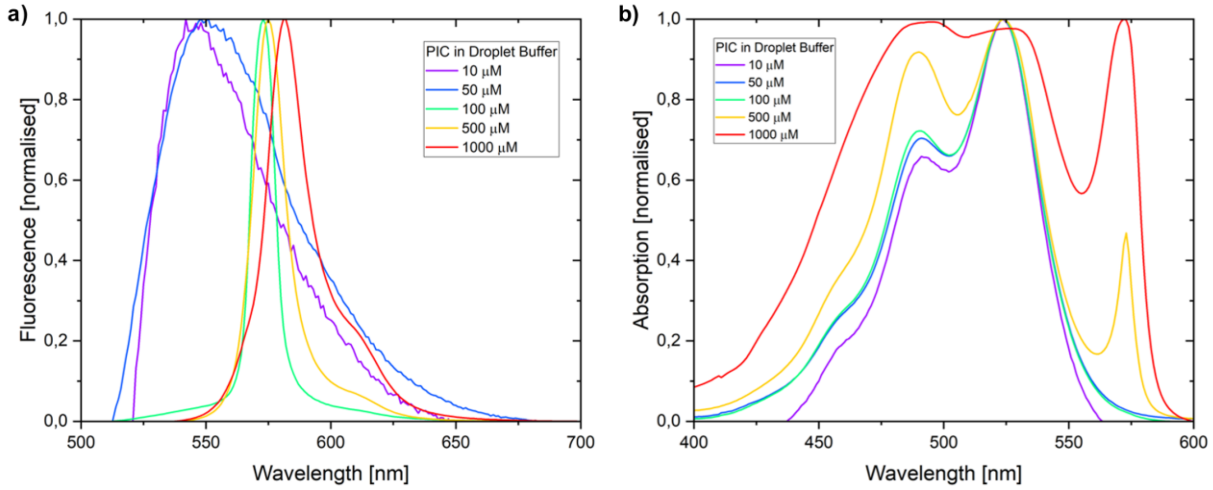

Supplementary Fig. 5. Spectra of PIC in droplet buffer at various PIC concentrations. a) Monomeric PIC was observed for 10 and 50 µM, higher concentrations of PIC showed the narrow peak of the J-aggregate (100 to 1000 µM). b) The absorption spectra of PIC showed for the concentration from 10 to 100 µM the spectrum of the monomeric PIC, while the narrow peak of the J-aggregate at 573 nm was apparent at 500 and 1000 µM.

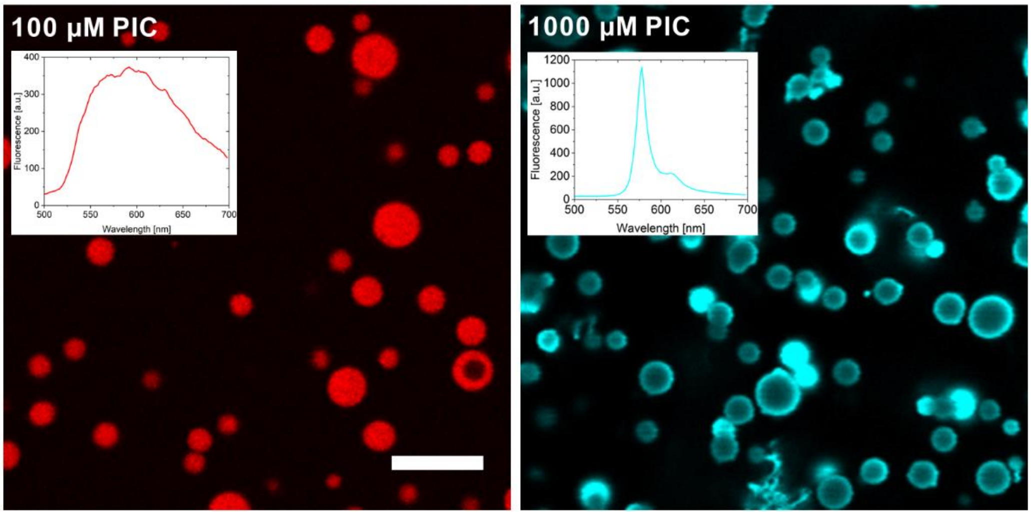

Supplementary Fig. 6. PIC solution at a concentration of 100 µM (monomeric PIC) and 1000 µM (aggregated PIC) was added to matured droplets at 21°C. *Left:* PIC monomer was enriched within droplets (*inset:* fluorescence spectrum of PIC in the droplets). *Right:* PIC aggregates accumulated on the surface of the droplets after addition to the solution (*inset:* fluorescence spectrum of PIC aggregates). The scale bar represents 10 µm.

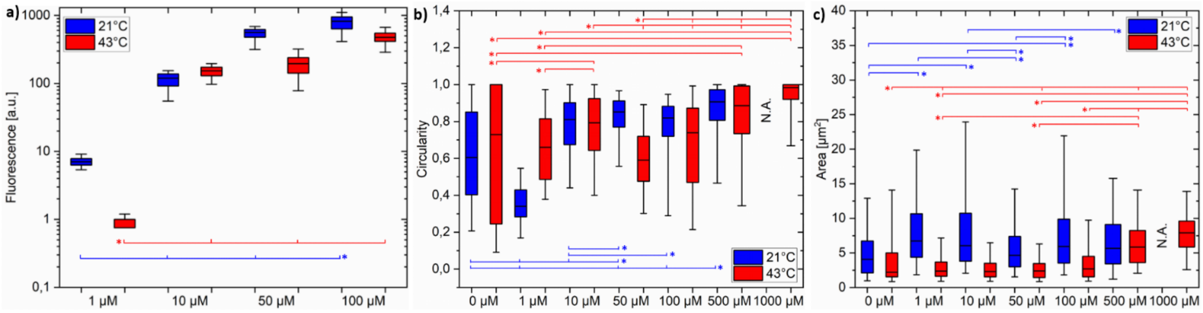

Supplementary Fig. 7. Fluorescence intensity (a), circularity (b), and area (c) of FUS droplets at 21°C and 43°C and different PIC concentrations. At each concentration over 150 droplets were evaluated. ANOVA analysis revealed significance ($p<0.05$) in the asterisks marked cases. The whiskers represent 5-95% of all data points. Details in Supplementary Table 3.

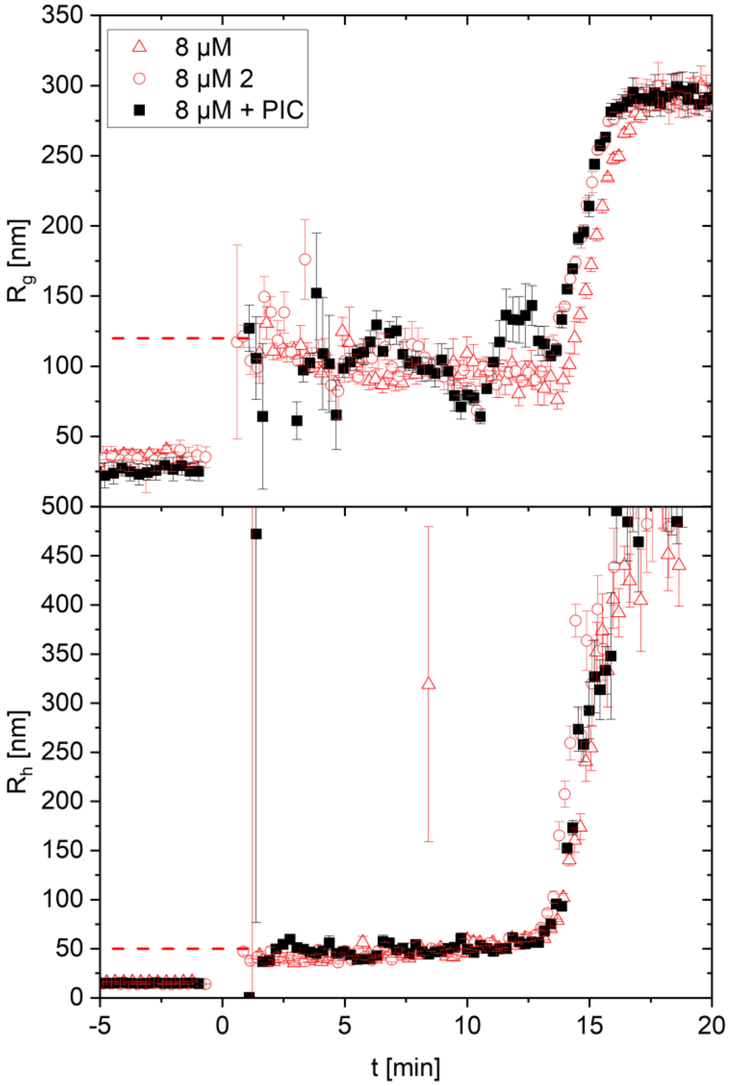

Supplementary Fig. 8. Evolution of the radius of gyration R_g and hydrodynamic radius R_h before and during the liquid-liquid phase separation of 8 µM MBP-FUS without PIC (Δ ,O) and with 50 µM PIC (■) in buffer triggered by adding MBP-FUS into 0.1 mg/l TEV. The red dashed lines represent the radius of gyration and hydrodynamic radius of pure FUS in buffer. Data points at the negative time represent TEV in buffer.

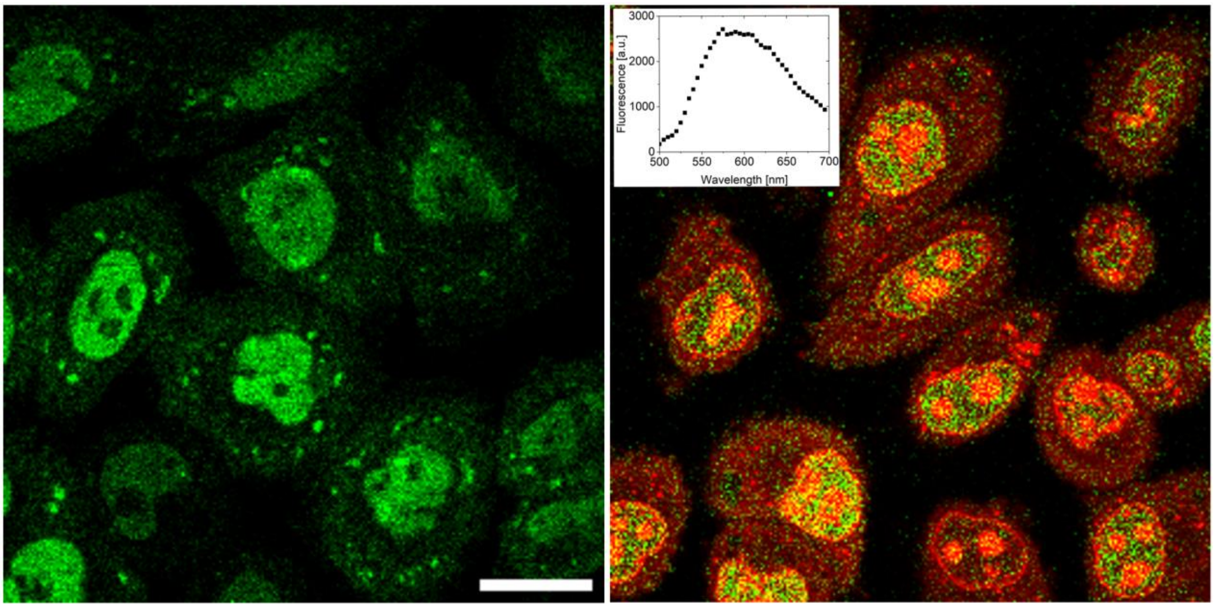

Supplementary Fig. 9. HeLa$^{FUS\text{-}GFP}$ cells stressed by 1 mM sodium arsenate for 1 h at 37°C *(left)*. Incubation with PIC and arsenate for 30 min led to monomeric PIC in the cells (*right*, fluorescence spectrum shown as inset). Scale bar represents 20 µm.

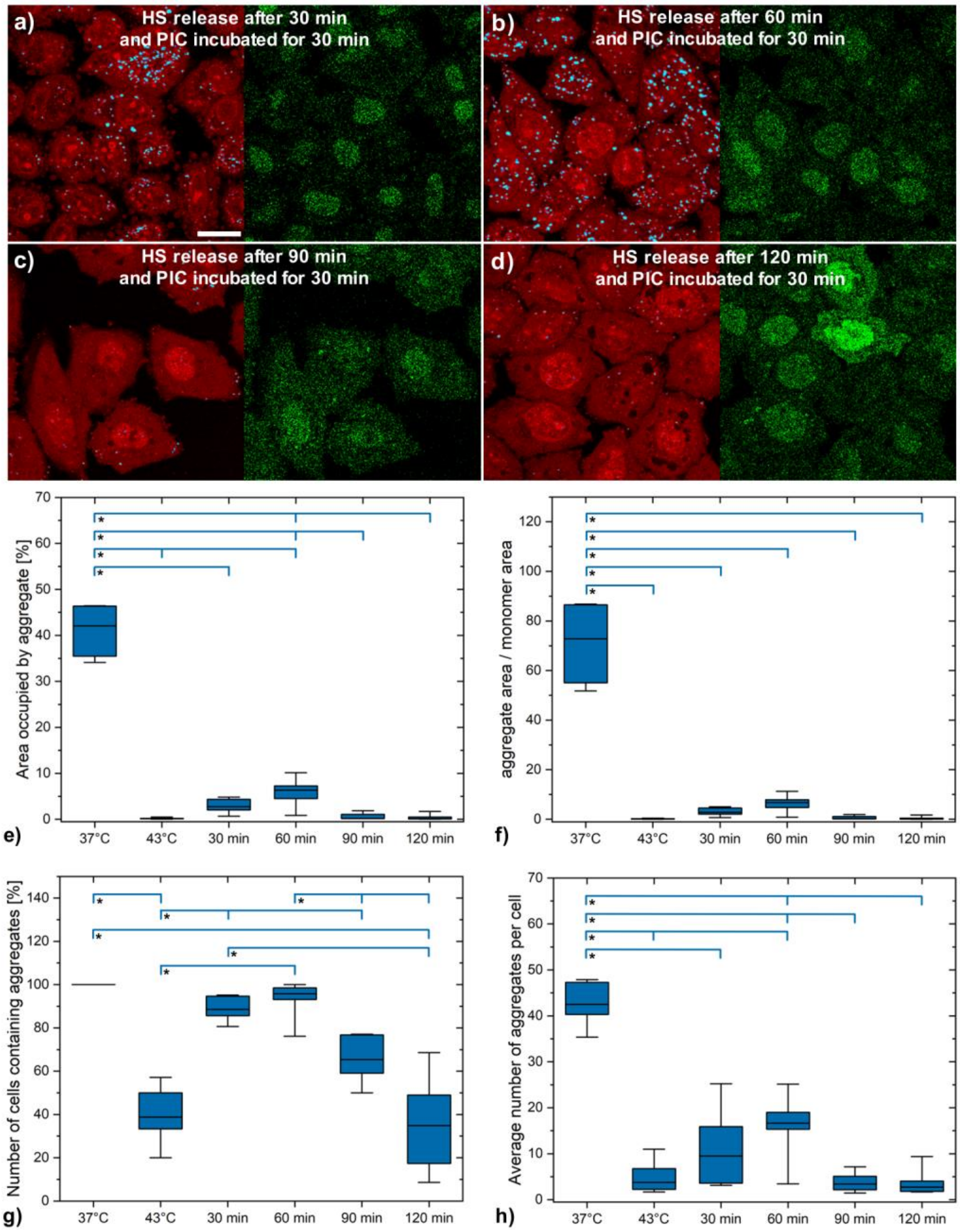

Supplementary Fig. 10. HeLa$^{FUS\text{-}GFP}$ cells were treated with different durations of HS (30, 60, 90 and 120 min) and afterwards recovered at 37°C for 30 min. a-d) PIC aggregates (blue) and monomers (red) were assigned based on their intensity and spectrum (see Methods section "Confocal laser scanning microscopy" for details). *Left images*: PIC monomer (red), J-aggregate (blue); *Right images*: FUS-GFP (green). PIC was added after HS and incubated at 37°C for 30 min. The scale bar represents 20 µm. e-h) Cell population statistics for the exemplary images shown in a-d) analysing the area occupied by PIC aggregates in the cytoplasm (e), aggregate area divided by monomer area (f), number of cells containing aggregates (g) and average number of aggregates per cell (h). The results were compared to physiological conditions (37°C) and HS (43°C) without recovery to 37°C. ANOVA analysis revealed significance ($p<0.05$) in the asterisks marked cases. The whiskers represent 5-95% of all data points. Details in Supplementary Table 4.

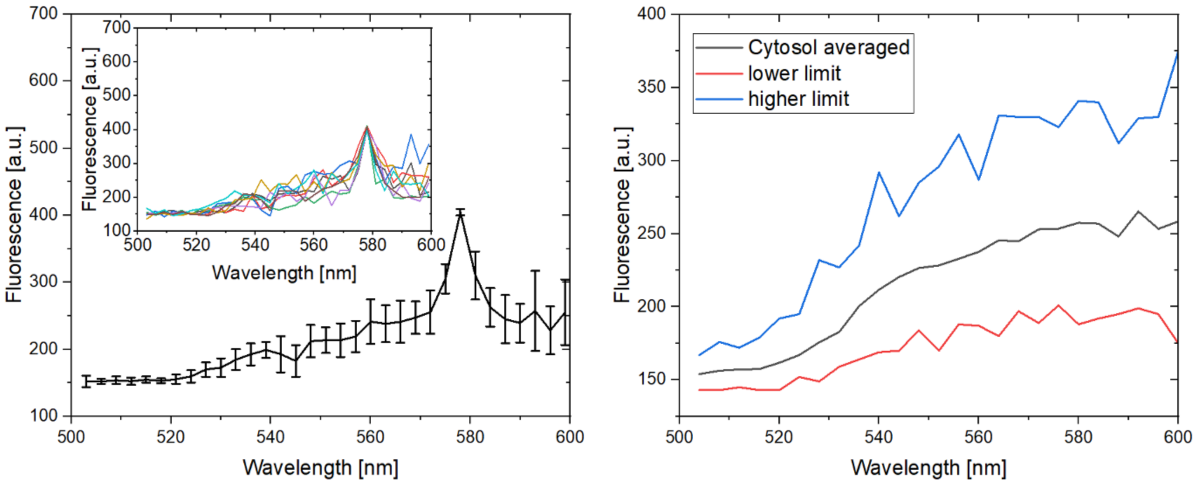

Supplementary Fig. 11. Threshold of fluorescence intensity to distinguish between the monomer and J-aggregate of PIC. Left: The peak at 575 nm indicated J-aggregate. Inset: Intensities above 400 were unambiguously attributed to J-aggregates. Right: Average fluorescence intensity (black) of the monomer with the higher (blue) and lower (red) fluorescence limit.

Supplementary Table 1. Count of cells with SGs and co-localisation of PIC and vice versa at 43°C (Figure 1). The measurements were conducted at 5 independent days including 10 samples from a single cell batch each day.

Sample #	Cell with SGs	FUS SGs count	Co-loc. with PIC accumulations [%]	Count PIC accumulations	Co-loc. with FUS SGs [%]	Area occupied by SGs [%]
1	10	28	82	151	15	1,0
2	8	40	72	379	8	1,2
3	5	40	60	103	23	2,8
4	6	14	64	76	12	1,1
5	7	18	44	93	8	1,6
6	6	38	66	169	15	4,1
7	4	22	50	66	17	1,9
8	5	58	38	171	13	1,9
9	6	21	67	86	16	1,7
10	5	29	59	64	27	2,8
11	6	29	48	146	10	2,0
12	6	20	60	159	7	2,7
13	9	29	59	220	8	2,5
14	5	36	31	84	13	2,8
15	10	36	42	260	6	1,3
16	8	29	72	250	8	2,9
17	10	47	40	111	17	2,4
18	8	31	42	86	16	2,0
19	7	38	58	328	6	1,8
20	9	18	94	162	10	3,8
21	8	61	46	264	11	2,2
22	7	53	53	316	9	1,8
23	5	18	44	125	6	2,2
24	7	46	61	290	10	1,6
25	8	34	62	314	6	1,5
26	6	12	75	157	5	5,1
27	7	26	58	157	10	1,9
28	10	44	73	418	7	6,5
29	10	48	56	280	9	3,6
30	8	35	51	268	7	5,5
31	8	28	75	346	5	5,2
32	5	31	68	274	8	3,3
33	5	34	56	181	10	2,6
34	6	32	53	241	7	4,4
35	5	30	67	159	13	1,8
36	3	30	67	143	14	3,4
37	6	12	58	162	4	3,5
38	6	29	79	399	6	2,4
39	4	22	77	149	11	1,8
40	10	20	55	130	8	1,5
41	7	44	70	328	9	3,4
42	6	17	71	242	5	1,6
43	6	30	63	298	6	1,3
44	3	21	52	99	11	1,3
45	9	26	50	254	5	3,6
46	10	23	78	311	6	3,2
47	9	28	86	219	11	3,1
48	6	17	59	133	8	4,7
49	7	29	45	125	10	2,0
50	9	24	67	242	6	3,7

Supplementary Table 2. Two-sample t-test results on SG properties (Figure 1d-f). N=10 for FRAP experiments and N=50 for circularity and area.

Reference	p-value
Mobile fraction (Figure 1d)	0.00577
Circularity (Figure 1e)	< 0.0001
Area (Figure 1f)	0.00408

Supplementary Table 3. One-way ANOVA calculated the significance for Supplementary Fig. 7. At each condition at least 150 droplets were evaluated, from 3 technical replicates.

References	Fluorescence 21°C (Supplementary Fig. a)	Fluorescence 43°C (Supplementary Fig. a)	Circularity 21°C (Supplementary Fig. b)	Circularity 43°C (Supplementary Fig. b)	Area 21° (Supplementary Fig. c)	Area 43°C (Supplementary Fig. c)
0 µM to 1 µM	NA	NA	<0,0001	0,9867	<0,0001	0,1262
0 µM to 10 µM	NA	NA	<0,0001	<0,0001	<0,0001	0,0232
0 µM to 50 µM	NA	NA	<0,0001	0,7255	0,2157	0,0993
0 µM to 100 µM	NA	NA	<0,0001	0,7603	<0,0001	0,9756
0 µM to 500 µM	NA	NA	<0,0001	<0,0001	0,1296	<0,0001
0 µM to 1000 µM	NA	NA	NA	<0,0001	NA	0
1 µM to 10 µM	<0,0001	NA	<0,0001	<0,0001	0,9146	0,9823
1 µM to 50 µM	<0,0001	NA	<0,0001	0,1306	<0,0001	0,9993
1 µM to 100 µM	<0,0001	NA	<0,0001	0,09965	0,6803	0,3180
1 µM to 500 µM	NA	NA	<0,0001	<0,0001	0,0693	<0,0001
1 µM to 1000 µM	NA	NA	NA	<0,0001	NA	0
10 µM to 50 µM	<0,0001	0	<0,0001	<0,0001	<0,0001	0,9999
10 µM to 100 µM	<0,0001	0	0,8741	<0,0001	0,8433	0,0527
10 µM to 500 µM	NA	NA	0,0011	0,1206	0,0196	<0,0001
10 µM to 1000 µM	NA	NA	NA	<0,0001	NA	0
50 µM to 100 µM	<0,0001	0	<0,0001	0,0011	<0,0001	0,2602
50 µM to 500 µM	NA	NA	0,5408	<0,0001	0,8517	<0,0001
50 µM to 1000 µM	NA	NA	NA	0	NA	0
100 µM to 500 µM	NA	NA	<0,0001	<0,0001	0,1195	<0,0001
100 µM to 1000 µM	NA	NA	NA	0	NA	0
500 µM to 1000 µM	NA	NA	NA	<0,0001	NA	<0,0001

Supplementary Table 4. One way ANOVA calculated significances for Supplementary Fig. 10.

References	Area occupied by aggregates (Supplementary Fig. a)	Aggregate area / monomer area (Supplementary Fig. b)	Number of cells containing aggregates (Supplementary Fig. c)	Average aggregates per cell (Supplementary Fig. d)l
43°C to 37°C	<0,0001	<0,0001	<0,0001	<0,0001
30 min to 37°C	<0,0001	<0,0001	0,6278	<0,0001
30 min to 43°C	0,5474	0,9784	<0,0001	0,0846
60 min to 37°C	<0,0001	<0,0001	0,8279	<0,0001
60 min to 43°C	0,0145	0,6201	<0,0001	0,0008
60 min to 30 min	0,4289	0,9528	0,9895	0,0435
90 min to 37°C	<0,0001	<0,0001	0,0011	<0,0001
90 min to 43°C	0,9997	0,9999	0,0182	0,9998
90 min to 30 min	0,7738	0,9929	0,0439	0,1833
90 min to 60 min	0,0435	0,8386	0,0110	0,0029
120 min to 37°C	<0,0001	<0,0001	<0,0001	<0,0001
120 min to 43°C	0,9999	1	0,9943	1
120 min to 30 min	0,6983	0,9891	<0,0001	0,1165
120 min to 60 min	0,0321	0,7069	<0,0001	0,0016
120 min to 90 min	0,9999	1	0,0075	0,9999

Supplementary Table 5. Average of the ratio of R_g to R_h during droplet formation of 8 mg/l MBP-FUS in the absence and presence of 50 µM PIC.

Sample	R_g/R_h during droplet formation
8 mg/l MBP-FUS	0,71
8 mg/l MBP-FUS 2	0,82
8 mg/l MBP-FUS + PIC	0,93
