# Supplementary material for: Cell stress and phase separation stabilize the monomeric state of pseudoisocyanine chloride employed as a self-assembly crowding sensor
Source: Commun Chem. 2024 Oct 7;7:230. doi: 10.1038/s42004-024-01315-y (PMC11458801; doi:10.1038/s42004-024-01315-y)
Supplement: Supplementary file 3 — Description of Additional Supplementary Files [file 42004_2024_1315_MOESM3_ESM.pdf]

Description of Additional Supplementary Files

File name: Supplementary Data 1

Description: Raw data for fluorescence spectra presented in figures 2b-c, 4 and Supplementary figures 2, 3, 4, 5,6 and 9 as well as the absorption spectra in supplementary figure 1
